# Ambient Air Pollutant Exposures and Hospitalization for Kawasaki Disease in Taiwan: A Case-Crossover Study (2000–2010)

**DOI:** 10.1289/EHP137

**Published:** 2016-07-26

**Authors:** Chau-Ren Jung, Wei-Ting Chen, Yu-Ting Lin, Bing-Fang Hwang

**Affiliations:** 1 Department of Occupational Safety and Health, and; 2 Department of Public Health, College of Public Health, China Medical University, Taichung, Taiwan; 3 Department of Atmospheric Science, National Taiwan University, Taipei, Taiwan

## Abstract

**Background:**

Kawasaki disease (KD) is an acute and multi-systemic vasculitis that occurs predominantly in infants and young children. Although the etiological agent of KD remains unclear, limited studies have reported that windborne environmental factors may trigger KD.

**Objectives:**

We conducted a time-stratified case-crossover study to assess the associations between air pollutants and KD in Taiwan.

**Methods:**

We identified children < 5 years old with a diagnosis of KD from the Longitudinal Health Insurance Database 2000 (LHID2000) between 2000 and 2010. We obtained data regarding carbon monoxide (CO), nitrogen dioxide (NO_2_), ozone (O_3_), particulate matter with aerodynamic diameter < 10 μm (PM_10_), and sulfate dioxide (SO_2_) from 70 monitoring stations and used inverse distance weighting to calculate average daily exposures for the residential postal code of each case. We performed conditional logistic regression to estimate associations between KD and each air pollutant according to interquartile range (IQR) increases and quartiles of exposure on the day of hospitalization versus 3–4 reference days during the same month for each case. Additionally, we estimated associations with single-day exposures lagged 1–2 days.

**Results:**

We identified 695 KD hospital admissions during the study period. An IQR increase (28.73 ppb) of O_3_ was positively associated with KD after adjusting for temperature, humidity, northward wind, and eastward wind [adjusted odds ratio = 1.21; 95% confidence interval (CI): 1.01, 1.44]. There were no significant associations between KD and CO, NO_2_, PM_10_, or SO_2_. The association with O_3_ was limited to exposure on the day of hospitalization and to exposure during the summer months (June–August).

**Conclusions:**

Our results provide new evidence that exposure to O_3_ may increase the risk of KD in children. However, further investigation is needed to confirm the association and identify a potential biological mechanism.

**Citation:**

Jung CR, Chen WT, Lin YT, Hwang BF. 2017. Ambient air pollutant exposures and hospitalization for Kawasaki disease in Taiwan: a case-crossover study (2000–2010). Environ Health Perspect 125:670–676; http://dx.doi.org/10.1289/EHP137

## Introduction

Kawasaki disease (KD) is an acute and multi-systemic vasculitis that predominantly occurs in infants and young children < 5 years old. In approximately 15–25% of untreated cases, KD may affect the coronary arteries and lead to myocardial infarction (MI), sudden death, or ischemic heart disease in childhood or early adulthood (Burns and Glodé 2004; Newburger et al. 2004). The clinical features of the disease are fever for more than five days; bilateral bulbar conjunctival injection without exudates; cervical lymphadenopathy; changes in extremities with both erythema and induration; changes in lips and oral mucosa including erythema, cracking, strawberry tongue, and diffuse injection of oral and pharyngeal mucosa; and polymorphous rash (Newburger et al. 2004). Although several etiological theories have been proposed, including environmental toxin exposure, autoimmune pathogenesis, and infectious diseases, the etiology of KD remains unknown (Rowley 2011).

The incidence of KD has a striking seasonal variation, with different patterns in several countries (Belay et al. 2006; Burns et al. 2005; Chang et al. 2004; Du et al. 2007; Huang et al. 2009; Lynch et al. 2003; Ng et al. 2005; Park et al. 2007), as well as geographical and temporal clustering (Kao et al. 2008). A study involving postmortem examinations demonstrated that the causal agent of KD entered through the upper respiratory tract, leading to a systemic immune response in vascular tissue, pancreas, and kidney (Rowley et al. 2000). Rodó and colleagues (2011) reported that an increase of KD cases in Japan, Hawaii, and San Diego was associated with a large-scale shift in the Asia-North Pacific wind pattern, further suggesting that the etiological agent of KD is spread by wind. Further research conducted by this group suggested that the cause of KD in Japan may be an agent carried by tropospheric winds from the densely cultivated region of northeastern China (Rodó et al. 2014). In addition, the authors concluded that the temporal pattern of KD cases is not consistent with an infectious etiology given evidence of a very short lag between exposure and the onset of symptoms (< 1 day) (Rodó et al. 2014).

Ambient air contains a mixture of individual pollutants, such as free radicals [e.g., nitrogen dioxide (NO_2_)], and pollutants that have the ability to trigger free radical reactions [e.g., ozone (O_3_) and particulate matter]. Exposure to elevated air pollutants can stimulate oxidative stress, induce inflammation in the lungs and the vascular system and cause subsequent responses that are particularly dangerous to susceptible individuals (Kelly 2003). To the best of our knowledge, only one study has assessed the associations between air pollutants and KD. In their analysis of KD in Japan, Rodó and colleagues (2014) also evaluated exposures to sulfur dioxide (SO_2_), oxidants (O_x_), nitrogen oxides (NO_x_), carbon monoxide (CO), and nonmethane hydrocarbon (measured at three stations in the Tokyo Metropolitan area) as potential causes of KD but reported that the results were negative (Rodó et al. 2014). Whether air pollutants contribute to KD is still unclear owing to a lack of consideration for possible confounding factors and limited monitoring data. We therefore conducted a case-crossover study to assess the associations between short-term air pollutants and KD after controlling for meteorological variables.

## Methods

### Data Source

The data used in the present study were sourced from the Longitudinal Health Insurance Database 2000 (LHID2000). LHID2000 is a representative subset of data that includes data from 1,000,000 individuals systematically and randomly selected from the year 2000 registry of beneficiaries of the Taiwan National Health Insurance Research Database (NHIRD) (NHRI 2016). NHIRD was established in March 1995 and includes detailed information, such as outpatient visits, hospital admissions, prescriptions, and diagnosis of disease, based on the *International Classification of Diseases, Ninth Revision, Clinical Modification* (ICD-9-CM) (NCHS 2017), from 99% of the entire population of 23 million enrollees in Taiwan. The National Health Insurance Administration (NHI) verifies the validity and quality of diagnosis by randomly sampling a constant ratio of claims from every hospital each year and through strict review by an independent group of medical experts (Lin et al. 2010). The accuracy of diagnosis of major diseases, such as acute coronary syndrome and ischemic stroke, has been validated (Cheng et al. 2011; Wu et al. 2010). Besides, the NHI has classified KD as a catastrophic illness. In Taiwan, individuals who are diagnosed with a catastrophic illness receive a certificate that provides outpatient or inpatient care that is free of charge. The diagnosis of KD is assigned by a board-certified specialist and must be reviewed and approved by NHI; therefore, the diagnosis of KD is accurate and reliable (Wei et al. 2014). The National Health Research Institutes confirmed that there are no significant differences in the gender distribution, age distribution, number of newborns every year, and average insurance payroll amount between the beneficiaries of LHID2000 and NHIRD. Because the data were analyzed anonymously, the institute review board specifically waived the need for consent from each subject. This study has been approved by the Institute Review Board of China Medical University Hospital, and it complies with the principles outlined in the Helsinki Declaration.

### Outcome of Interest

We identified children < 5 years old who had a diagnosis of KD (ICD-9-CM code 446.1) from 1 January 2000 to 31 December 2010. To ensure the diagnostic validity of KD in the present study, only cases of KD where the ICD-9-CM code was assigned by a pediatrician were selected as our outcome of interest (Kuo et al. 2014). The index date of the KD cases was set as the date of hospital admission. We extracted 1,453 hospital admissions with a diagnosis of KD (ICD-9-CM code 446.1) from the LHID2000. We excluded patients who were ≥ 5 years old (*n* = 756) and patients with missing postcodes (*n* = 2).

For depicting the demographic characteristics of KD cases, municipal-level household income was accessed from annual average incomes data assessed by the Taiwan Directorate-General of Budget, Accounting and Statistics. We divided Taiwan main island into four regions according to administrative divisions: northern, central, southern, and eastern (Figure S1).

### Exposure Assessment

We obtained hourly data of CO, NO_2_, SO_2_, particulate matter with aerodynamic diameter < 10 μm (PM_10_) and O_3_ from 70 monitoring stations constructed by Taiwan Environmental Protection Administration (TEPA) on Taiwan’s main island that provided measurements continuously from 2000 to 2010 (TEPA 2016). Methods used for measuring these pollutants were non-dispersive infrared absorption for CO, chemiluminescence for NO_2_, ultraviolet absorption for O_3_, beta-gauge for PM_10_, and ultraviolet fluorescence for SO_2_. These data were subjected to rigorous quality assurance and control procedures through independent projects. TEPA authorized an independent private sector to perform annual performance audits and regular performance checks for monitoring instruments (TEPA 2010).

For each monitoring station, the daily average of air pollutants [24 hr for CO, NO_2_, SO_2_, and PM_10_; 8 hr (1000–1800 hours) for O_3_] were calculated for subsequent analyses. At least 75% of the 1-hr values had to be available for the days included in this calculation. In addition, these 70 monitoring stations were classified into five types: 54 general stations, 4 industrial stations, 6 traffic stations, 2 national park stations, and 4 background stations. When daily average data of air pollutants for a monitoring station were not available, we used the average value from the other monitoring stations of the same type within the same day to fulfill missing values.

The locations of the monitoring stations and air pollution sources were identified and managed by ArcGIS, a geographic information system (version 10; ESRI, Redlands, CA, USA). The monitoring data were interpolated to pollutant surfaces using the inverse distance weighting (IDW) method. For the IDW approach, we used suitable spatial resolution (100 m) (Stroh et al. 2007) and the inverse squared distance (1/squared distance) weighted average of the three nearest monitors within 50 km of each grid cell (the study area was divided into 100 m × 100 m grid cells) to compute the daily average concentration for each air pollutant. Then, the daily air pollution data were assigned to individuals according to the residential postcode on the day of hospitalization. The average spatial resolution of postcodes in Taiwan was 17 ± 8.56 km^2^ in urban areas but was larger in rural areas (154 ± 104.39 km^2^) with low-population density. For cross-validation of models, we randomly selected data from 63 monitoring stations (90% of 70 monitoring stations) to estimate air pollution using the IDW model and then retained seven stations (10%) for evaluation.

### Meteorological Variables

We used meteorological values from the European Center for Medium-Range Weather Forecasts (ECMWF)-Interim data set (ECMWF 2015), a global atmospheric re-analysis from 1979 to 2016 produced by the ECMWF (Dee et al. 2011). We extracted the following values from 2000 to 2010: temperature at 2 m above the ground; dew point temperature at 2 m above the ground; eastward wind at 10 m above the ground (zonal wind or U wind); and northward wind at 10 m above the ground (meridional wind or V wind), with a spatial resolution of 0.125° longitude × 0.125° latitude in a nested grid covering Taiwan. We used values recorded at Coordinated Universal Time (UTC) 0000, 0600, 1200, and 1800 hours every day to calculate the daily average of these variables. The value of temperature and dew point temperature at 2 m above the ground from the same four time points were used to calculate average relative humidity for each day. Owing to the moist environment in Taiwan (relative humidity > 50%), we used the following simple formula to compute relative humidity:

RH ≈ 100 – 5(*t* – *t_d_*)

where RH is the relative humidity in percent, and *t_d_* and *t* are the dew point temperature at 2 m above the ground and temperature at 2 m above the ground in degrees Celsius, respectively (Lawrence 2005). Wind in the atmosphere can be represented mathematically by a vector to declare speed and direction and be decomposed into two components: eastward wind, whose positive value means wind flows from west to east; and northward wind, whose positive value means wind flows from south to north. The average daily values of meteorological variables were also assigned to individuals according to the postcodes.

### Study Design

We used a time-stratified case-crossover design to investigate the associations between exposure to air pollutants and the risk of KD. The case-crossover design is the most widely used design for investigating acute health effects of air pollution (Carracedo-Martínez et al. 2010). In this design, each individual serves as his or her own control by using the exposure on the days before or after the case day. Owing to the consistency of the subject across both days, this design can control for the influence of confounding factors such as gender, smoking history, occupational history and genetics (Maclure 1991). The time-stratified approach was proposed to avoid bias from season and day of the week by restricting the reference days to the same day of the week within the same month and year as the case day (the day of hospitalization for KD) (Janes et al. 2005). For example, if a KD case was admitted to the hospital on the first Monday of January 2006 (2 January 2006), all other Mondays within January 2006 were assigned as the reference days for that case (9, 16, 23, and 30 January 2006). This approach resulted in 3 or 4 reference days for each case.

### Statistical Analyses

We used Spearman’s correlation to examine the relationships between air pollutants and meteorological variables. Conditional logistic regression was used to estimate the associations between air pollutant and KD, with results reported as odds ratios (ORs) per interquartile range (IQR) with their 95% confidence intervals (CI) (PROC PHREG, version 9.4; SAS Institute, Cary, NC, USA). We first fitted single pollutant models to estimate the association of exposure to an individual air pollutant with KD. If there was a statistically significant association (*p*-value < 0.05) between an individual pollutant and KD, we then examined the robustness of the association between that specific pollutant and KD after controlling for other pollutants. If the correlation coefficient between two pollutants was greater than 0.5, we did not include it in the same model to avoid collinearity. We also compared the association of four exposure categories based on the quartiles of the distribution of an individual pollutant (high: > 75th percentile; medium: 75th to 50th percentile; low: 50th to 25th percentile; and reference: < 25th percentile) with the risk of KD. The chi-square test for linear trend in binomial proportions was applied to test whether there was an increasing trend in the proportion of KD for exposure categories. In addition to estimating associations with exposures on the day of hospitalization (lag 0), we estimated associations with single-day lagged exposures on the previous day (lag 1) and 2 days before hospitalization (lag 2). To assess any seasonal pattern, we conducted analyses for single pollutants stratified by season (spring: March–May; summer: June–August; autumn: September–November; winter: December–February). Daily mean values of temperature at 2 m above the ground, relative humidity, eastward wind at 10 m above the ground, and northward wind at 10 m above the ground were included in all models as simple continuous covariates to adjust for potential confounding. Additionally, we conducted sensitivity analyses to compare ORs with and without adjustment for eastward and northward wind.

## Results

A total of 695 KD hospital admissions were identified from our study population from 1 January 2000 to 31 December 2010. The mean age at admission was 2.54 years, ranging from 0.11 to 4.99 years and most cases were male (56.83%) (Table 1). Approximately one-third of the children hospitalized for KD lived in municipalities with a high-household income (> $1,158,981 NTD; 34.10%). Most KD admissions were from the southern, northern, and western regions of Taiwan (37.55%, 36.69%, and 23.74%, respectively); only a few were from the eastern region of Taiwan (2.01%) (Table 1).

**Table 1:** Demographic data of Kawasaki disease (KD) admissions (*n* = 695, age 2.54 ± 1.21 years) from 1 January 2000 to 31 December 2010 in Taiwan.

Variables	Number of events (%)
Sex
Female	300 (43.17)
Male	395 (56.83)
Household income
< $897,000 NTD	198 (28.49)
$897,000–1,095,192 NTD	136 (19.57)
$1,095,192–1,158,981 NTD	124 (17.84)
> $1,158,981 NTD	237 (34.10)
Region
Northern	255 (36.69)
Central	165 (23.74)
Southern	261 (37.55)
Eastern	14 (2.01)
Note: Municipal-level household income of KD admissions were accessed from annual average incomes data assessed by the Taiwan Directorate-General of Budget, Accounting and Statistics. The regions of Taiwan were divided according to administrative divisions. NTD, new Taiwan dollar.	

### Air Pollution and Meteorological Variables

The IDW model performances were high for O_3_ and PM_10_ (*R^2^* = 0.62 for O_3_; *R^2^* = 0.78 for PM_10_) and low for CO, NO_2_, and SO_2_ (*R^2^* = 0.20 for CO; *R^2^* = 0.32 for NO_2_; *R^2^* = 0.35 for SO_2_) (Table S1). The distributions of daily average concentrations of air pollutants and meteorological variables by using time-stratified case-crossover design in Taiwan during the study period (2000–2010) are presented in Table 2. Annual average values of daily mean CO and NO_2_ concentrations decreased during 2000–2010 (Table S2 and Figure S2). Annual average values of daily mean O_3_ concentration increased steeply from 2000 to 2004 (from 40.14 ppb to 47.45 ppb), and then reached a plateau from 2005 to 2010 (fluctuated between 43.57 ppb and 48.07 ppb) (Table S2).

**Table 2:** Distribution of daily data of air pollutants and meteorological variables using time-stratified case-crossover design in Taiwan, 2000–2010.

Daily environmental variable	Mean ± SD	Minimum	Q1	Q2	Q3	Maximum	IQR
Air pollutants
CO (ppm)	0.75 ± 0.35	0.15	0.54	0.69	0.88	9.27	0.34
NO_2_ (ppb)	23.20 ± 9.41	2.64	16.02	22.33	29.36	72.02	13.34
O_3_ (ppb)	42.75 ± 19.36	1.17	27.18	41.13	55.91	106.52	28.73
PM_10_ (μg/m^3^)	57.39 ± 29.88	10.75	34.54	50.62	75.14	235.43	40.60
SO_2_ (ppb)	4.43 ± 2.80	0.05	2.40	3.92	5.87	25.68	3.47
Meteorological variable
Temperature (°C)	21.86 ± 5.03	5.54	18.14	22.89	25.97	30.69	7.83
Humidity (%)	81.85 ± 8.67	51.15	75.86	82.38	88.36	98.98	12.50
U10 (m/sec)	–1.10 ± 2.02	–10.76	–2.46	–1.19	0.19	7.95	2.65
V10 (m/sec)	–0.72 ± 2.19	–9.88	–2.26	–0.75	0.86	8.60	3.12
Note: CO, carbon monoxide; IQR: interquartile range; NO_2_, nitrogen dioxide; O_3_, ozone; PM_10_, particulate matter with aerodynamic diameter < 10 μm; Q1, 25th percentile; Q2, 50th percentile; Q3, 75th percentile; SD, standard deviation; SO_2_, sulfur dioxide; U10, eastward wind at 10 m above the ground; V10, northward wind at 10 m above the ground.							

Table 3 shows the Spearman’s correlation coefficients between daily air pollutant concentrations and meteorological variables. The daily average concentration of CO was highly and positively correlated with NO_2_ (*r* = 0.77) and moderately correlated with PM_10_ (*r* = 0.48). The concentration of PM_10_ was highly and positively correlated with NO_2_ (*r* = 0.63), and moderately correlated with O_3_ (*r* = 0.57) and SO_2_ (*r* = 0.54). The northward wind was moderately correlated with temperature (*r* = 0.52) and eastward wind (*r* = 0.45). In general, the daily air pollutant concentrations were negatively correlated with humidity (*r* ranged from –0.01 to –0.51) (Table 3).

**Table 3:** Spearman’s correlation between daily average air pollutants and meteorological variables in Taiwan, 2000–2010.

	CO	NO_2_	O_3_	PM_10_	SO_2_	Temperature	Humidity	U10
CO	1.00
NO_2_	0.77**	1.00
O_3_	0.14**	0.23**	1.00
PM_10_	0.48**	0.63**	0.57**	1.00
SO_2_	0.40**	0.55**	0.27**	0.54**	1.00
Temperature	–0.23**	–0.43**	0.05*	–0.30**	0.02	1.00
Humidity	–0.01	–0.17**	–0.51**	–0.43**	–0.29**	–0.05*	1.00
U10	–0.03	–0.11**	–0.10**	–0.14**	–0.01	0.39**	0.11**	1.00
V10	–0.07**	–0.22**	–0.09**	–0.21**	–0.00	0.52**	–0.01	0.45**
Note: CO, carbon monoxide; NO_2_, nitrogen dioxide; O_3_, ozone; PM_10_, particulate matter with aerodynamic diameter equal to or less than 10 μm; SO_2_, sulfur dioxide; U10, eastward wind at 10 m above the ground; V10, northward wind at 10 m above the ground. *p < 0.05. **p < 0.001.								

The correlations between municipal-level household income and air pollution were weak in Taiwan (*r* = –0.13 for O_3_; *r* = 0.28 for CO; *r* = 0.13 for NO_2_; *r* = –0.09 for PM_10_; *r* = 0.01 for SO_2_).

### Associations between Air Pollution and KD

**Figure 1:**
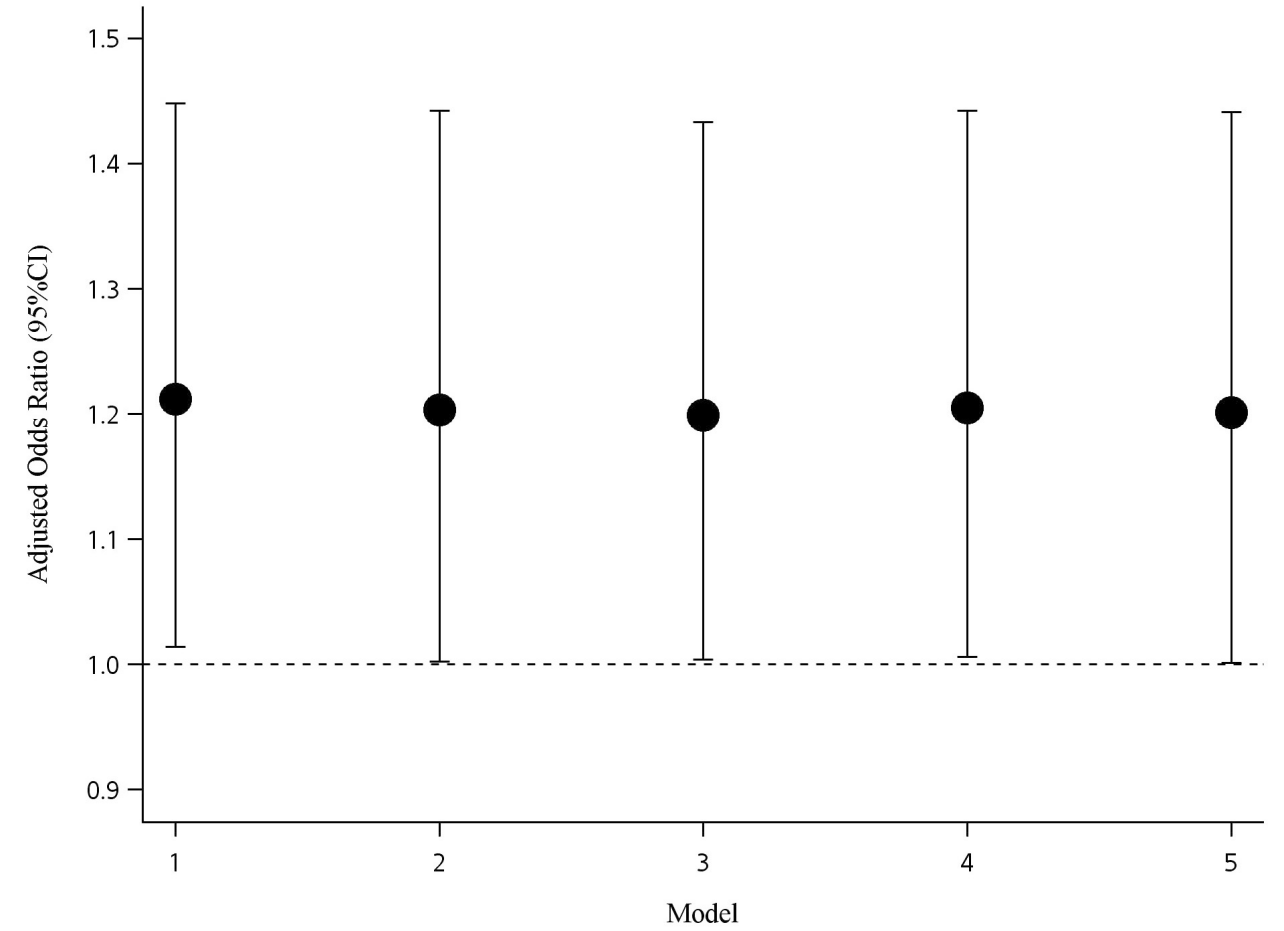
Associations between Kawasaki disease and ozone from two- and three-pollutant models in Taiwan, 2000–2010. The associations are shown as odds ratio (OR) with 95% confidence interval (CI) for an interquartile range (28.73 ppb) increase in ozone. All models were adjusted for temperature, humidity, northward wind, and eastward wind. Model 1 included ozone and carbon monoxide. Model 2 included ozone and nitrogen dioxide. Model 3 included ozone and sulfur dioxide. Model 4 included ozone, sulfur dioxide, and carbon monoxide. Model 5 included ozone, sulfur dioxide, and nitrogen dioxide. Note: CI, confidence interval.

In the single pollutant model, an IQR increase (per 28.73 ppb change) of O_3_ concentration was positively associated with KD (adjusted OR = 1.21; 95% CI: 1.01, 1.44) (Table 4). The second (27.18–41.13 ppb) and fourth (55.91–106.52 ppb) quartiles of O_3_ exposure were significantly associated with KD when compared with the lowest quartile of exposure, but the OR for the third quartile was not significant, and we did not observe a clear linear trend when O_3_ was modeled as a categorical variable (Table 4). The *p*-value for trend was significant for NO_2_ modeled as a categorical variable, but the association was positive (but non-significant) for the highest quartile only (Table 4). There were no significant associations between KD and CO, PM_10_, or SO_2_ (Table 4). The association between KD and an IQR increase in O_3_ remained stable and significant after adjusting for the other pollutants in two- and three-pollutant models (adjusted OR varying between 1.19 and 1.21) (Figure 1). However, we did not evaluate two- and three-pollutant models for associations with pollutants other than O_3_, because the other pollutants were not significantly associated with KD in single-pollutant models.

**Table 4:** Associations between Kawasaki disease and air pollution from single pollutant models in Taiwan, 2000–2010.

Air pollutants	OR (95% CI)	Adjusted OR^*a*^	*p* for trend^*b*^
O_3_
per 28.73 ppb change	1.17 (1.01, 1.35)	1.21 (1.01, 1.44)
1st Quartile^*c*^, 1.17–27.18 ppb	1.00	1.00	0.11
2nd Quartile, 27.18–41.13 ppb	1.27 (0.99, 1.63)	1.30 (1.00, 1.68)
3rd Quartile, 41.13–55.91 ppb	1.23 (0.95, 1.59)	1.28 (0.97, 1.70)
4th Quartile, 55.91–106.52 ppb	1.33 (1.00, 1.75)	1.40 (1.01, 1.94)
NO_2_
per 13.34 ppb change	1.05 (0.87, 1.26)	1.08 (0.89, 1.30)
1st Quartile^*c*^, 2.64–16.02 ppb	1.00	1.00	0.02
2nd Quartile, 16.02–22.33 ppb	0.99 (0.75, 1.30)	0.98 (0.74, 1.29)
3rd Quartile, 22.33–29.36 ppb	1.00 (0.72, 1.39)	0.99 (0.71, 1.38)
4th Quartile, 29.36–72.02 ppb	1.24 (0.87, 1.76)	1.28 (0.90, 1.83)
CO
per 0.34 ppm change	0.99 (0.89, 1.11)	1.01 (0.91, 1.13)
1st Quartile^*c*^, 0.15–0.54 ppm	1.00	1.00	0.06
2nd Quartile, 0.54–0.69 ppm	0.91 (0.70, 1.19)	0.92 (0.70, 1.19)
3rd Quartile, 0.69–0.88 ppm	1.03 (0.77, 1.37)	1.04 (0.78, 1.40)	
4th Quartile, 0.88–9.27 ppm	1.12 (0.81, 1.54)	1.20 (0.86, 1.66)
PM_10_
per 40.60 μg/m^3^ change	1.10 (0.94, 1.28)	1.10 (0.94, 1.30)
1st Quartile^*c*^, 10.75–34.54 μg/m^3^	1.00	1.00	0.22
2nd Quartile, 34.54–50.62 μg/m^3^	1.12 (0.87, 1.45)	1.11 (0.86, 1.45)
3rd Quartile, 50.62–75.14 μg/m^3^	1.01 (0.77, 1.33)	1.01 (0.75, 1.35)
4th Quartile, 75.14–235.43 μg/m^3^	1.17 (0.85, 1.60)	1.16 (0.83, 1.63)
SO_2_
per 3.47 ppb change	1.06 (0.92, 1.22)	1.06 (0.92, 1.23)
1st Quartile^*c*^, 0.05–2.40 ppb	1.00	1.00	0.69
2nd Quartile, 2.40–3.92 ppb	1.19 (0.91, 1.54)	1.18 (0.82, 1.55)
3rd Quartile, 3.92–5.87 ppb	1.09 (0.82, 1.45)	1.10 (0.82, 1.48)
4th Quartile, 5.87–25.68 ppb	1.13 (0.83, 1.55)	1.14 (0.82, 1.59)
Note: CO, carbon monoxide; NO_2_, nitrogen dioxide; O_3_, ozone; OR, odds ratio; PM_10_, particulate matter with aerodynamic diameter < 10 μm; SO_2_, sulfur dioxide. ^***a***^Adjusted ORs were adjusted for temperature, humidity, northward wind, and eastward wind. ^***b***^The *p* for trend was computed by using the chi-square test for linear trend in binomial proportions. ^***c***^Reference group.			

**Figure 2:**
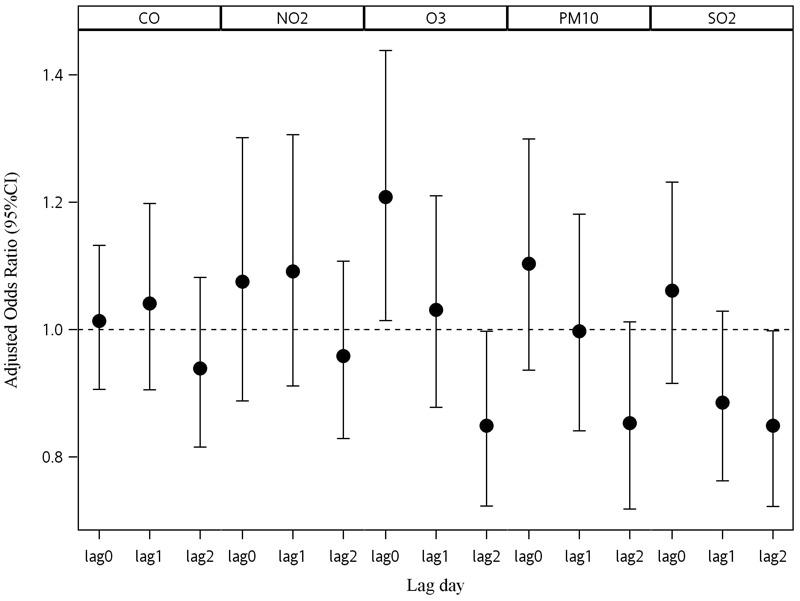
Associations between Kawasaki disease and air pollution in Taiwan, 2000–2010 at different lag days. The associations are shown as odds ratio (OR) with 95% confidence interval (CI) for interquartile range (IQR) increases in exposure (see Table 2 for the IQRs for each pollutant). All models were adjusted for temperature, humidity, northward wind, and eastward wind. Note: CO, carbon monoxide; NO_2_, nitrogen dioxide; O_3_, ozone; PM_10_, particulate matter with aerodynamic diameter < 10 μm; SO_2_, sulfur dioxide.

Figure 2 presents the lag pattern of air pollutants on KD. ORs for an IQR increase in CO and NO_2_ were slightly higher for exposure on the previous day (lag 1) than on the same day (lag 0), while ORs for the other pollutants were positive for exposure on lag0 (the current day), close to the null or negative (< 1.0) for exposure on the previous day (lag 1), and negative for exposure 2 days prior (lag 2) (Figure 2). The only significant positive association with KD was for O_3_ on the same day. KD showed a significant negative association with IQR increases in O_3_ and SO_2_ at lag day 2 (OR = 0.85; 95% CI: 0.72, 1.00 and OR = 0.85; 95% CI: 0.72, 1.00, respectively).

**Figure 3:**
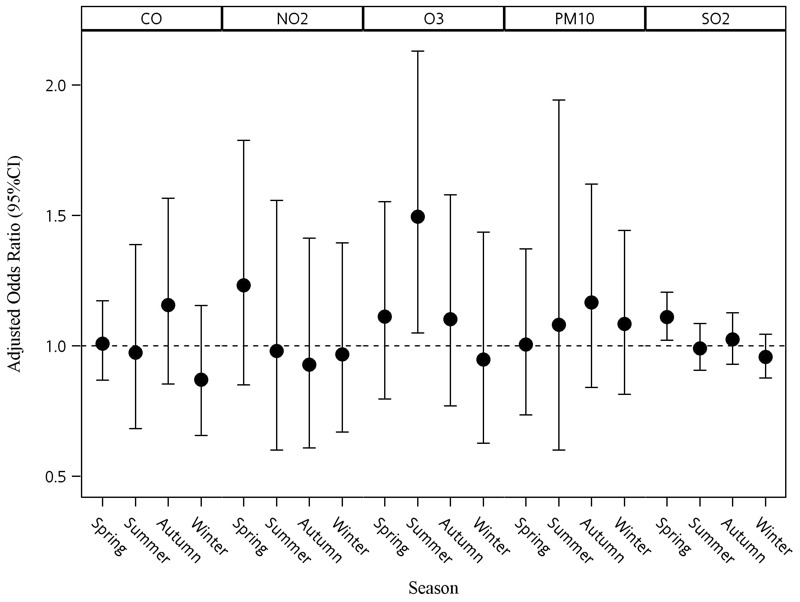
Associations between air pollutants and Kawasaki disease stratified by season. The associations are shown as odds ratio (OR) with 95% confidence intervals (CIs) for interquartile range (IQR) increase in exposure (the IQRs are based on overall distributions; see Table 2 for the IQRs for each pollutant). Models were adjusted for temperature, humidity, eastward wind, and northward wind. Autumn, September–November; CO, carbon monoxide; NO_2_, nitrogen dioxide; O_3_, ozone; PM_10_, particulate matter with aerodynamic diameter < 10 μm; SO_2_, sulfur dioxide; Spring, March–May; Summer, June–August; Autumn, September–November; Winter, December–February.

Figure 3 shows the associations between air pollutants and KD stratified by season. For each pollutant, we used the same IQR value for all seasons. The association between O_3_ and KD was significant in summer (adjusted OR = 1.50, 95% CI: 1.05, 2.13 per 28.73 ppb), while the associations of O_3_ for all other seasons were close to the null (Figure 3). In addition, the association between SO_2_ and KD was significant in spring (adjusted OR = 1.11, 95% CI: 1.02, 1.21 per 3.47 ppb change).

We also evaluated whether the eastward and northward wind at 10 m above the ground might influence associations between air pollutants and KD. The ORs from model with and without adjusted for eastward and northward wind did not change more than 10% (see Table S3).

## Discussion

In this study, we used a time-stratified case-crossover study to investigate the associations between air pollutants and KD. We estimated that an IQR increase (28.73 ppb) in O_3_ concentration was associated with a 21% increase in the odds of hospitalization for KD (95% CI: 1, 44%). Thanks at least in part to more stringent air quality regulations and emission controls, there has been a long-term decrease in the traffic-related air pollutants CO and NO_2_ (10.6 ppb and 0.21 ppb decrease per year) in Taiwan (Chen et al. 2015). Contrary to these gaseous pollutants, the concentration of O_3_ has gradually increased (0.83 ppb increase per year) from 1994 to 2010 in Taiwan (Chen et al. 2015), which might represent a great environmental health problem. The increasing concentration of O_3_ in western Taiwan reflects the economic growth in East Asia over the last few decades (Chen et al. 2015).

In the present study, we did not find a monotonic trend for the association between quartiles of O_3_ with KD, though the association was strongest for the highest versus lowest quartile, positive for the second and third quartiles (though the OR for the third quartile was slightly lower than that for the second, and was not significant), and significant for an IQR increase in O_3_ modeled as a continuous variable. After adjusting for temperature, humidity, northward wind and eastward wind, the significant negative associations of KD with O_3_ and SO_2_ exposures 2 days before hospitalization (lag 2, Figure 2) suggests a pattern consistent with short-term displacement (or “harvesting effect”) (Zanobetti and Schwartz 2008), whereby the children who are vulnerable to KD as a consequence of exposure develop symptoms on the day of exposure, leaving no vulnerable children left to develop KD 2 days later. However, this pattern needs to be confirmed in other study populations.

Although the mean O_3_ concentration over Taiwan is lower during the summer than at other times of the year due to the elevated mixing layer height, unstable atmospheric conditions, and the formation of clouds or showers in afternoon during the summer (Cheng et al. 2015; Chou et al. 2006; Tsai et al. 2008), the association between O_3_ and KD appeared to be specific to exposures during the summer months (June–August). The observed results could be explained by the fact that children spend more time outside in summer, which in turn could increase personal exposure levels, and there may be other explanations as well (e.g., higher infiltration of O_3_ through open windows in the summer than in other seasons). However, additional research will be required to confirm the seasonal pattern and investigate potential mechanisms.

Few studies have assessed the association between air pollution and KD. Rodó and colleagues postulated a fungus, *Candida*, as a possible factor that may be carried by wind from the cultivated region of northeastern China to Japan (Rodó et al. 2014). Data on the presence or amount of *Candida* in ambient air was not available, but we adjusted for eastward and northward wind as an indirect means of controlling for potential confounding by fungus exposures in our analyses. Adjusting for both eastward and northward wind had little influence on associations (Table S3), which indicates that these variables were not strong confounders in our study. However, it is not possible for us to rule out potential confounding or effect modification by *Candida* exposure.

KD is an acute vasculitis that predominantly occurs in young children and particularly affects the coronary arteries (Brown et al. 2001; Burns and Glodé 2004; Newburger et al. 2004). Rowley and colleagues (2000) found a striking inflammation in the trachea of patients with KD. The progression of arterial lesions in KD involves perivasculitis of small arteries from days 0 to 9; panvasculitis of medium-sized, muscular arteries with aneurysm formation and thrombosis from days 12 to 25; coronary and other medium-sized artery myointimal proliferation from days 28 to 31; and narrowing of the arteries may occur after 40 days (Burns et al. 2000). Exposure to O_3_ may enhance the release of inflammatory mediators from the airway epithelial cells and exacerbate the risk of adverse health effects in susceptible individuals (Bayram et al. 2001). Even in a developing primate infant, episodic exposure to O_3_ can result in the loss of a number of terminal bronchioles, a reduction of distal airway size and alterations in smooth muscle bundle orientation (Fanucchi et al. 2006). Acute and chronic lung inflammation due to pollutants can result in systemic inflammation that triggers a cascade of events outside the lung and then causes cardiovascular disease (Srebot et al. 2009; Tamagawa and van Eeden 2006). Chuang and colleagues (2009) used three different animal models (wild-type C57Bl/6 mice, apoE^–/–^ mouse, and infant macaque monkey) and found that inhalation of O_3_ increased vascular dysfunction, oxidative stress and mitochondrial damage and, in apoE^–/–^ mouse, accelerated atherogenesis.

Several epidemiological studies have also reported associations between cardiovascular disease and O_3_. Breton and colleagues (2012) conducted a cross-sectional study with a cohort of 768 American college students that showed that exposure to a 9.3-ppb increase in O_3_ during elementary school years (ages 6–12) was associated with a 10.1 μm (95% CI: 1.8, 18.5) higher carotid artery intima-media thickness in young adults. A case-crossover study involving 635 acute MI cases in France suggested that short-term exposure to a 5-ppb increase in O_3_ on 2 consecutive days was related to a 5% increased risk of acute MI in French middle-aged adults [5% (95% CI: 1, 8% for the current day (lag 0) and 5% (95% CI: 1, 9% for the previous day (lag 1), respectively)] (Ruidavets et al. 2005). A time series study conducted in Denver, one of the most polluted cities in the United States, reported that exposure to an IQR change (9.7 ppb) in O_3_ was positively associated with an increase in the risk of hospitalization for coronary atherosclerosis and pulmonary heart disease [12.3% (95% CI: 4.0, 21.4%) and 21.4% (95% CI: 4.0, 41.8%), respectively)] (Koken et al. 2003). In a large meta-analysis involving 144 estimates from 39 time-series studies, Bell and colleagues (2005) found that a 10-ppb increase in O_3_ was associated with a 2.45% (95% CI: 0, 4.10%) increase in cardiovascular mortality during the warm season. By investigating 23 European cities with mean number of cardiovascular disease deaths per day ranging from 2 to 143 involved in Air Pollution and Health: a European Approach project (APHEA2), Gryparis and colleagues (2004) found a significant association between a 10-μg/m^3^ increases in 1-hr O_3_ and cardiovascular mortality in the warm season [0.45% (95% CI: 0.22, 0.69%)].

Our study had some potential limitations. First, air pollutant data were accessed from ambient air pollution monitoring stations rather than being measured at personal exposure levels and could not represent indoor concentration of air pollutants. We used the IDW method in this study to estimate air pollution concentration based on postcode rather than personal address, which may have increased the possibility of random exposure misclassification. The IDW method is a more appropriate approach for secondary pollutants that vary on a larger geographical scale and are more homogeneously distributed (e.g., O_3_) than for those that are more localized and dependent on combustible sources (e.g., CO, NO_2_) (Vrijheid et al. 2011). In this study, we consistently found that the IDW model performed better for O_3_ and PM_10_ than for other pollutants (Table S1). The differences in associations between KD and the different pollutants is partly a function of the accuracy of exposure estimation. Second, possible confounders in studying the association of air pollution with KD include genetic factors, infectious disease (e.g., staphylococcus infection, super antigens), fungal toxins, and socioeconomic factors, among others. Personal invariant factors such as genetic factors were controlled for using the time-stratified case-crossover design. Although possible confounding by socioeconomic factors could not be completely ruled out in our study, we did not observe a strong correlation between air pollution and municipal-level household income in Taiwan. The universal insurance program (NHIRD) in Taiwan was launched in 1995 and currently covers almost all residents (99%) (NHI 2012); therefore, children’s access to health services may be less influenced by socioeconomic factors in Taiwan than in some other countries. Additionally, Chang and colleagues (2013) conducted a case–control study included 115 KD cases and 1,150 controls in Taiwan from 1997 to 2010 to assess the association between KD and urbanization. They used population density, percentage of people with college-level education or higher, percentage of elderly > 65 years old, percentage of agricultural workers in the population, and number of physicians per 100,000 individuals in the population to classify urbanization and indicated that the association between urbanization and KD was not statistically significant. Unfortunately, we could not exclude confounding by infectious disease and fungal toxins due to a lack of data. Third, the index date (date of hospital admission) was the only available information on the timing of the KD event in our data source. The date of hospital admission does not represent the true timing of disease onset, which may have led to exposure misclassification. Although residents in Taiwan have variety of choice for seeking health care without financial barriers, easy access to the physicians and specialists, and short waiting times for health care services (Cheng 2015), the bias due to time mismatch between hospital admission and true timing of disease onset in our study was inevitable.

## Conclusion

Our results provide new evidence that children exposed to O_3_ may have an increased risk of hospitalization for KD. However, further investigation is needed to confirm our findings and evaluate potential biological mechanisms.

## Supplemental Material

(836 KB) PDF

## References

[r1] Bayram H, Sapsford RJ, Abdelaziz MM, Khair OA (2001). Effect of ozone and nitrogen dioxide on the release of proinflammatory mediators from bronchial epithelial cells of nonatopic nonasthmatic individuals and atopic asthmatic patients in vitro.. J Allergy Clin Immunol.

[r2] Belay ED, Maddox RA, Holman RC, Curns AT, Ballah K, Schonberger LB (2006). Kawasaki syndrome and risk factors for coronary artery abnormalities: United States, 1994–2003.. Pediatr Infect Dis J.

[r3] Bell ML, Dominici F, Samet JM (2005). A meta-analysis of time-series studies of ozone and mortality with comparison to the National Morbidity, Mortality, and Air Pollution Study.. Epidemiology.

[r4] Breton CV, Wang X, Mack WJ, Berhane K, Lopez M, Islam TS (2012). Childhood air pollutant exposure and carotid artery intima-media thickness in young adults.. Circulation.

[r5] Brown TJ, Crawford SE, Cornwall ML, Garcia F, Shulman ST, Rowley AH (2001). CD8 T lymphocytes and macrophages infiltrate coronary artery aneurysms in acute Kawasaki disease.. J Infect Dis.

[r6] Burns JC, Cayan DR, Tong G, Bainto EV, Turner CL, Shike H (2005). Seasonality and temporal clustering of Kawasaki syndrome.. Epidemiology.

[r7] Burns JC, Glodé MP (2004). Kawasaki syndrome.. Lancet.

[r8] Burns JC, Kushner HI, Bastian JF, Shike H, Shimizu C, Matsubara T (2000). Kawasaki disease: a brief history.. Pediatrics.

[r9] Carracedo-Martínez E Taracido M Tobias A Saez M Figueiras A 2010 Case-crossover analysis of air pollution health effects: a systematic review of methodology and application. Environ Health Perspect 118 1173 1182, doi:10.1289/ehp.0901485 20356818 PMC2920078

[r10] Chang LY, Chang IS, Lu CY, Chiang BL, Lee CY, Chen PJ (2004). Epidemiologic features of Kawasaki disease in Taiwan, 1996–2002.. Pediatrics.

[r11] Chang WP Wu SJ Chang WC Kuo HC 2013 Population-based study of the association between urbanization and Kawasaki disease in Taiwan. ScientificWorldJournal 2013 169365, doi:10.1155/2013/169365 23864819 PMC3706024

[r12] Chen JP, Yang CE, Tsai IC (2015). Estimation of foreign versus domestic contributions to Taiwan’s air pollution.. Atmos Envrion.

[r13] Cheng CL, Kao YH, Lin SJ, Lee CH, Lai ML (2011). Validation of the National Health Insurance Research Database with ischemic stroke cases in Taiwan.. Pharmacoepidemiol Drug Saf.

[r14] Cheng FY, Jian SP, Yang ZM, Yen MC, Tsuang BJ (2015). Influence of regional climate change on meteorological characteristics and their subsequent effect on ozone dispersion in Taiwan.. Atmos Environ.

[r15] Cheng TM (2015). Reflections on the 20th anniversary of Taiwan’s single-payer National Health Insurance System.. Health Aff (Millwood).

[r16] Chou CCK, Liu SC, Lin CY, Shiu CJ, Chang KH (2006). The trend of surface ozone in Taipei, Taiwan, and its causes: implications for ozone control strategies.. Atmos Environ.

[r17] Chuang GC, Yang Z, Westbrook DG, Pompilius M, Ballinger CA, White CR (2009). Pulmonary ozone exposure induces vascular dysfunction, mitochondrial damage, and atherogenesis.. Am J Physiol Lung Cell Mol Physiol.

[r18] Dee DP, Uppala SM, Simmons AJ, Berrisford P, Poli P, Kobayashi S (2011). The ERA-Interim reanalysis: configuration and performance of the data assimilation system.. Q J R Meteorol Soc.

[r19] Du ZD, Zhao D, Du J, Zhang YL, Lin Y, Liu C (2007). Epidemiologic study on Kawasaki disease in Beijing from 2000 through 2004.. Pediatr Infect Dis J.

[r20] ECMWF (European Centre for Medium-Range Weather Forecasts) (2015). ERA-Interim.. http://www.ecmwf.int/en/research/climate-reanalysis/era-interim.

[r21] Fanucchi MV, Plopper CG, Evans MJ, Hyde DM, Van Winkle LS, Gershwin LJ (2006). Cyclic exposure to ozone alters distal airway development in infant rhesus monkeys.. Am J Physiol Lung Cell Mol Physiol.

[r22] Gryparis A, Forsberg B, Katsouyanni K, Analitis A, Touloumi G, Schwartz J (2004). Acute effects of ozone on mortality from the “Air Pollution and Health: A European Approach” project.. Am J Respir Crit Care Med.

[r23] Huang WC, Huang LM, Chang IS, Chang LY, Chiang BL, Chen PJ (2009). Epidemiologic features of Kawasaki disease in Taiwan, 2003–2006.. Pediatrics.

[r24] Janes H, Sheppard L, Lumley T (2005). Case-crossover analyses of air pollution exposure data: referent selection strategies and their implications for bias.. Epidemiology.

[r25] Kao AS, Getis A, Brodine S, Burns JC (2008). Spatial and temporal clustering of Kawasaki syndrome cases.. Pediatr Infect Dis J.

[r26] Kelly FJ (2003). Oxidative stress: its role in air pollution and adverse health effects.. Occup Environ Med.

[r27] Koken PJ Piver WT Ye F Elixhauser A Olsen LM Portier CJ 2003 Temperature, air pollution, and hospitalization for cardiovascular diseases among elderly people in Denver. Environ Health Perspect 111 1312 1317, doi:10.1289/ehp.5957 12896852 PMC1241612

[r28] Kuo HC, Wu CM, Chang WP, Kuo CN, Yeter D, Lin CY (2014). Association between Kawasaki disease and autism: a population-based study in Taiwan.. Int J Environ Res Public Health.

[r29] Lawrence MG (2005). The relationship between relative humidity and the dew point temperature in moist air: a simple conversion and applications.. Bull Am Meteorol Soc.

[r30] Lin HC, Chen YH, Lee HC, Lin HC (2010). Increased risk of acute myocardial infarction after acute episode of schizophrenia: 6 year follow-up study.. Aust N Z J Psychiatry.

[r31] Lynch M, Holman RC, Mulligan A, Belay ED, Schonberger LB (2003). Kawasaki syndrome hospitalizations in Ireland, 1996 through 2000.. Pediatr Infect Dis J.

[r32] Maclure M (1991). The case-crossover design: a method for studying transient effects on the risk of acute events.. Am J Epidemiol.

[r33] NCHS (National Center for Health Statistics) (2017). ICD-9-CM Files via FTP 2000. *International Classification of Diseases, Ninth Revision, Clinical Modification* (ICD-9-CM).. https://www.cdc.gov/nchs/icd/icd9cm.htm.

[r34] Newburger JW, Takahashi M, Gerber MA, Gewitz MH, Tani LY, Burns JC (2004). Diagnosis, treatment, and long-term management of Kawasaki disease: a statement for health professionals from the Committee on Rheumatic Fever, Endocarditis and Kawasaki Disease, Council on Cardiovascular Disease in the Young, American Heart Association.. Circulation.

[r35] Ng YM, Sung RY, So LY, Fong NC, Ho MH, Cheng YW (2005). Kawasaki disease in Hong Kong, 1994 to 2000.. Hong Kong Med J.

[r36] NHI (National Health Insurance Administration) (2012). Universal Health Coverage in Taiwan.. http://www.nhi.gov.tw/Resource/webdata/21717_1_20120808UniversalHealthCoverage.pdf.

[r37] NHRI (National Health Research Institutes) (2016). Data Subsets: Longitudinal Health Insurance Database (LHID).. http://nhird.nhri.org.tw/en/Data_Subsets.html#S3.

[r38] Park YW, Han JW, Park IS, Kim CH, Cha SH, Ma JS (2007). Kawasaki disease in Korea, 2003–2005.. Pediatr Infect Dis J.

[r39] Rodó X Ballester J Cayan D Melish ME Nakamura Y Uehara R 2011 Association of Kawasaki disease with tropospheric wind patterns. Sci Rep 1 152, doi:10.1038/srep00152 22355668 PMC3240972

[r40] Rodó X, Curcoll R, Robinson M, Ballester J, Burns JC, Cayan DR (2014). Tropospheric winds from northeastern China carry the etiologic agent of Kawasaki disease from its source to Japan.. Proc Natl Acad Sci U S A.

[r41] Rowley AH (2011). Kawasaki disease: novel insights into etiology and genetic susceptibility.. Annu Rev Med.

[r42] Rowley AH, Shulman ST, Mask CA, Finn LS, Terai M, Baker SC (2000). IgA plasma cell infiltration of proximal respiratory tract, pancreas, kidney, and coronary artery in acute Kawasaki disease.. J Infect Dis.

[r43] Ruidavets JB, Cournot M, Cassadou S, Giroux M, Meybeck M, Ferrières J (2005). Ozone air pollution is associated with acute myocardial infarction.. Circulation.

[r44] Srebot V Gianicolo EA Rainaldi G Trivella MG Sicari R 2009 Ozone and cardiovascular injury. Cardiovasc Ultrasound 7 30, doi:10.1186/1476-7120-7-30 19552797 PMC2706799

[r45] Stroh E Harrie L Gustafsson S 2007 A study of spatial resolution in pollution exposure modelling. Int J Health Geogr 6 19, doi:10.1186/1476-072X-6-19 17547740 PMC1892775

[r46] Tamagawa E, van Eeden SF (2006). Impaired lung function and risk for stroke: role of the systemic inflammation response?. Chest.

[r47] TEPA (Taiwan Environmental Protection Administration) (2010). Taiwan Air Quality Monitoring Station Quality Assurance Project [in Chinese]. EPA-98-FA11-03-A145.. http://epq.epa.gov.tw/EPQ_ResultDetail.aspx?proj_id=GYLJQEHXBN&recno=&document_id=6771#tab4.

[r48] TEPA (2016). History Data Download.. http://taqm.epa.gov.tw/taqm/en/YearlyDataDownload.aspx.

[r49] Tsai DH, Wang JL, Wang CH, Chan CC (2008). A study of ground-level ozone pollution, ozone precursors and subtropical meteorological conditions in central Taiwan.. J Environ Monit.

[r50] Vrijheid M Martinez D Manzanares S Dadvand P Schembari A Rankin J 2011 Ambient air pollution and risk of congenital anomalies: a systematic review and meta-analysis. Environ Health Perspect 119 598 606, doi:10.1289/ehp.1002946 21131253 PMC3094408

[r51] Wei CC, Lin CL, Kao CH, Liao YH, Shen TC, Tsai JD (2014). Increased risk of Kawasaki disease in children with common allergic diseases.. Ann Epidemiol.

[r52] Wu CY, Chan FK, Wu MS, Kuo KN, Wang CB, Tsao CR (2010). Histamine_2_-receptor antagonists are an alternative to proton pump inhibitor in patients receiving clopidogrel.. Gastroenterolgy.

[r53] Zanobetti A, Schwartz J (2008). Mortality displacement in the association of ozone with mortality: an analysis of 48 cities in the United States.. Am J Respir Crit Care Med.

