# Supplementary material for: Ambient Air Pollutant Exposures and Hospitalization for Kawasaki Disease in Taiwan: A Case-Crossover Study (2000–2010)
Source: Environ Health Perspect. 2016 Jul 26;125(4):670–6. doi: 10.1289/EHP137 (PMC5381970; doi:10.1289/EHP137)
Supplement: (836 KB) PDF [file EHP137.s001.acco.pdf]

Environ Health Perspect
DOI: 10.1289/EHP137

Note to readers with disabilities: *EHP* strives to ensure that all journal content is accessible to all readers. However, some figures and Supplemental Material published in *EHP* articles may not conform to 508 standards due to the complexity of the information being presented. If you need assistance accessing journal content, please contact ehp508@niehs.nih.gov. Our staff will work with you to assess and meet your accessibility needs within 3 working days.

Supplemental Material

Ambient Air Pollutant Exposures and Hospitalization for Kawasaki Disease in Taiwan: A Case-Crossover Study (2000-2010)

Chau-Ren Jung, Wei-Ting Chen, Yu-Ting Lin, and Bing-Fang Hwang

Table of Contents

Figure S1. Regions of Taiwan. The Taiwan main island was divided into four regions according to administrative divisions. The map was created using ArcGIS 10.0.
Figure S2. The distribution of daily average concentration of five air pollutants from 70 monitoring stations during 2000-2010. O_3 8hr, ppb; CO, ppm; NO_2, ppb; PM_{10}, μg/m^3; SO_2, ppb.
Table S1. Model performance parameters of Inverse distance weighting (IDW) model for carbon monoxide (CO), nitrogen dioxide (NO_2), ozone (O_3), particulate matter with aerodynamic diameter less than 10μm (PM_{10}), and sulfur dioxide (SO_2).
Table S2. Summary statistics of annual average values for daily mean concentrations of air pollution from 70 monitoring stations in Taiwan during 2000-2010.
Table S3. Adjusted Odds Ratio (95% confidence interval) for Kawasaki disease from single pollutant models: a comparison between with and without adjusted for wind components.

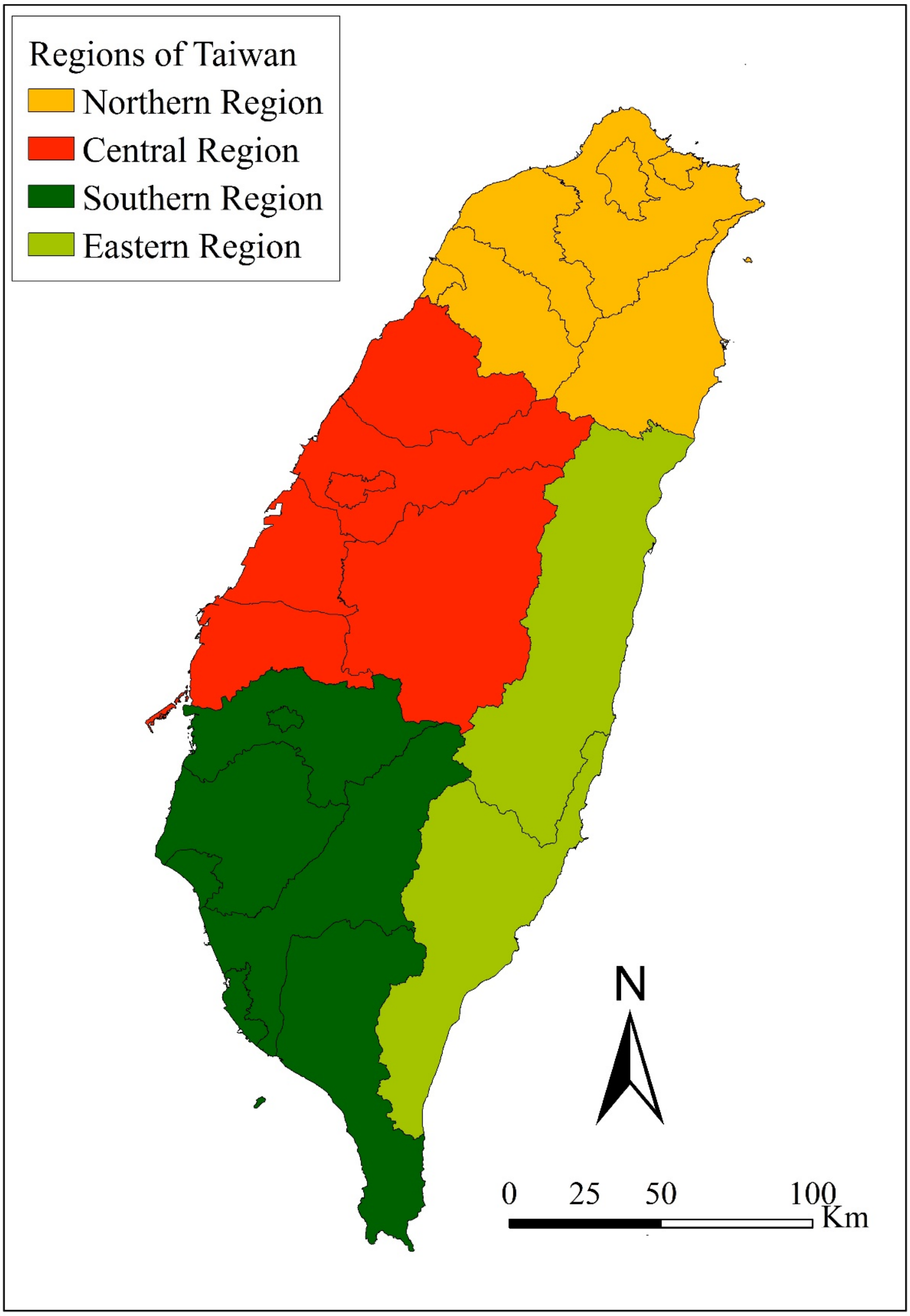

Figure S1. Regions of Taiwan. The Taiwan main island was divided into four regions according to administrative divisions. The map was created using ArcGIS 10.0.

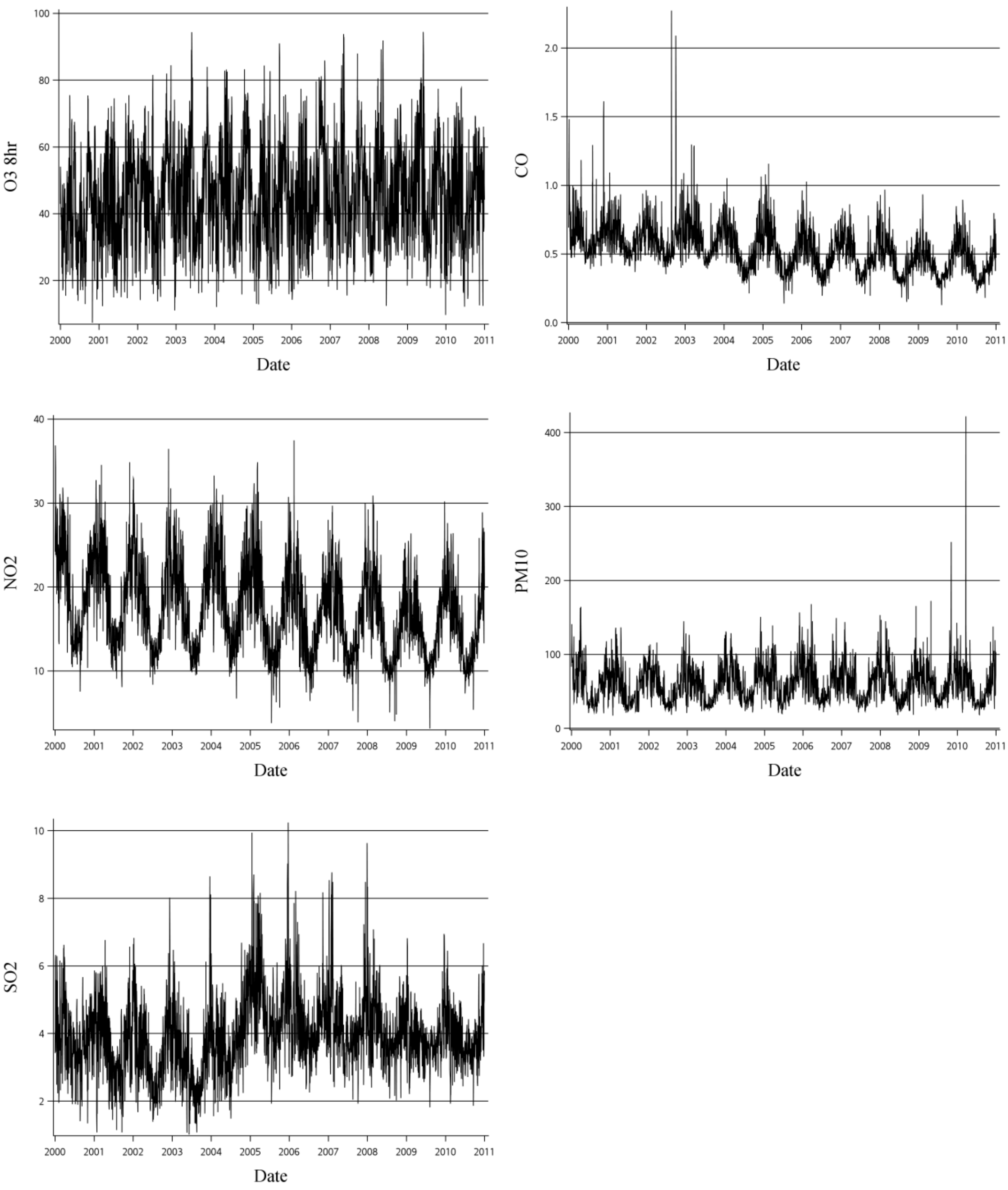

Figure S2. The distribution of daily average concentration of five air pollutants from 70 monitoring stations during 2000-2010. O_3 8hr, ppb; CO, ppm; NO_2, ppb; PM_{10}, $\mu g/m^3$; SO_2, ppb.

Table S1. Model performance parameters of Inverse distance weighting (IDW) model for carbon monoxide (CO), nitrogen dioxide (NO_2), ozone (O_3), particulate matter with aerodynamic diameter less than 10μm (PM_{10}), and sulfur dioxide (SO_2).

Pollutant	Coefficient of determination (R^2)	MAE	RMSE
CO	0.20	0.35	0.56
NO_2	0.32	8.22	12.14
O_3	0.62	8.95	11.76
PM_{10}	0.78	11.77	16.31
SO_2	0.35	1.74	2.41

Abbreviations: MAE, mean absolute error; RMSE, root mean square error.

For cross validation of models, we randomly selected data of 63 monitoring stations (90% of 70 monitoring stations) to estimate air pollution by IDW model, and then retained 7 stations (10%) for evaluation. Equations used to estimate R^2, MAE and RMSE are as below:

$$R^2 = 1 - \frac{\sum_{i=1}^{N}(O_i - E_i)^2}{\sum_{i=1}^{N}(O_i - \bar{O}_i)^2}$$

$$\text{MAE} = \frac{1}{N}\sum_{i=1}^{N}|E_i - O_i|$$

$$\text{RMSE} = \sqrt{\frac{1}{N}\sum_{i=1}^{N}(E_i - O_i)^2}$$

Where E, estimated value; O, observed value; N=number of observation; $\bar{O}$, Mean of observed values; i = 1, 2, …, N.

Table S2. Summary statistics of annual average values for daily mean concentrations of air pollution from 70 monitoring stations in Taiwan during 2000-2010.

Pollutants	Year	Average	Median	SD	Min	Max
	2000	40.14	37.89	18.47	0.38	117.31
	2001	43.53	41.45	19.03	1.14	129.49
	2002	45.54	43.54	19.59	1.17	127.90
	2003	45.96	44.28	18.99	2.23	131.40
	2004	47.45	45.13	19.95	1.69	127.00
O_3 8hr	2005	43.57	40.59	20.49	1.76	137.00
(ppb)	2006	46.40	43.14	20.33	8.66	133.17
	2007	46.85	44.10	20.15	2.44	148.20
	2008	46.14	43.46	18.90	2.46	126.00
	2009	48.07	45.35	19.91	4.30	134.40
	2010	44.73	43.13	17.72	3.68	115.20
	2000	0.65	0.59	0.50	0.06	37.33
	2001	0.62	0.57	0.26	0.03	3.54
	2002	0.62	0.55	0.74	0.06	49.38
	2003	0.62	0.57	0.41	0.04	25.88
	2004	0.54	0.50	0.24	0.00	4.03
CO	2005	0.52	0.48	0.25	0.01	3.53
(ppm)	2006	0.50	0.47	0.22	0.01	2.54
	2007	0.49	0.47	0.22	0.04	2.50
	2008	0.46	0.43	0.20	0.05	2.38
	2009	0.43	0.41	0.19	0.05	2.46
	2010	0.44	0.41	0.21	0.01	2.70
	2000	19.99	19.16	9.09	0.00	151.68
	2001	19.35	18.42	8.75	0.05	69.20
	2002	18.30	17.37	8.30	0.00	78.19
	2003	17.80	16.70	8.11	0.04	236.27
	2004	19.08	18.07	8.47	0.10	78.94
NO_2	2005	17.05	15.69	8.57	0.49	75.00
(ppb)	2006	16.73	15.73	8.01	0.47	67.79
	2007	16.44	15.32	7.88	0.42	70.20
	2008	15.57	14.48	7.60	0.76	60.00
	2009	15.10	14.25	6.85	0.59	54.45
	2010	15.61	14.54	7.43	0.40	65.03
	2000	61.18	51.01	37.20	10.90	306.65
	2001	57.58	49.83	31.78	6.87	245.41

	2002	55.55	49.19	29.69	11.04	354.02
	2003	56.51	50.75	28.30	9.25	205.00
	2004	63.79	56.67	32.42	4.41	301.60
PM_{10}	2005	64.14	57.65	34.13	3.77	233.40
(ppb)	2006	61.41	52.15	33.53	5.41	267.10
	2007	60.14	52.06	31.50	5.85	276.50
	2008	59.89	51.81	32.71	3.57	379.80
	2009	61.53	55.18	35.29	3.90	1144.90
	2010	58.12	48.92	41.94	2.35	864.00
	2000	3.85	3.18	3.21	0.00	85.17
	2001	3.64	3.09	2.84	0.00	41.48
	2002	3.43	2.86	3.01	0.00	136.51
	2003	3.18	2.69	2.57	0.00	66.73
	2004	3.86	3.41	2.56	0.00	47.79
SO_2	2005	4.86	4.20	2.92	0.33	35.20
(ppb)	2006	4.31	3.81	2.30	0.52	33.30
	2007	4.26	3.75	2.30	0.27	38.35
	2008	4.09	3.66	2.10	0.32	34.37
	2009	3.86	3.47	1.89	0.49	35.00
	2010	3.85	3.49	1.89	0.42	28.99

Table S3. Adjusted Odds Ratio (95% confidence interval) for Kawasaki disease from single pollutant models: a comparison between with and without adjusted for wind components.

	without adjusted for wind components[a]	with adjusted for wind components[b]
O_3		
per 28.73 ppb	1.26 (1.06, 1.50)	1.21 (1.01, 1.44)
<27.18	1.00	1.00
27.18-41.13	1.34 (1.03, 1.73)	1.30 (1.00, 1.68)
41.13-55.91	1.34 (1.01, 1.77)	1.28 (0.97, 1.70)
≥55.91	1.51 (1.09, 2.09)	1.40 (1.01, 1.94)
NO_2		
per 13.34 ppb	1.07 (0.88, 1.29)	1.08 (0.89, 1.30)
<16.02	1.00	1.00
16.02-22.33	0.99 (0.71, 1.31)	0.98 (0.74, 1.38)
22.33-29.36	1.01 (0.72, 1.40)	0.99 (0.71, 1.38)
≥29.36	1.27 (0.89, 1.81)	1.28 (0.90, 1.83)
CO		
per 0.34 ppm	1.00 (0.89, 1.13)	1.01 (0.91, 1.13)
<0.54	1.00	1.00
0.54-0.69	0.92 (0.70, 1.20)	0.92 (0.70, 1.19)
0.69-0.88	1.04 (0.78, 1.40)	1.04 (0.78, 1.40)
≥0.88	1.17 (0.84, 1.62)	1.20 (0.86, 1.66)
PM_{10}		
per 40.60 $\mu g/m^3$	1.13 (0.96, 1.33)	1.10 (0.94, 1.30)
<34.54	1.00	1.00
34.54-50.62	1.14 (0.88, 1.49)	1.11 (0.86, 1.45)
50.62-75.14	1.05 (0.78, 1.41)	1.01 (0.78, 1.35)
≥75.14	1.22 (0.87, 1.72)	1.16 (0.83, 1.63)
SO_2		
per 3.47 ppb	1.07 (0.92, 1.24)	1.06 (0.92, 1.23)
<2.40	1.00	1.00
2.40-3.92	1.2 (0.92, 1.57)	1.18 (0.82, 1.55)
3.92-5.87	1.12 (0.83, 1.50)	1.10 (0.82, 1.48)
≥5.87	1.16 (0.84, 1.61)	1.14 (0.82, 1.59)

[a] adjusted for temperature at 2 meters above the ground and humidity.

[b] adjusted for temperature at 2 meters above the ground, humidity, eastward wind and northward wind at 10 meters above the ground.
